# Evaluation of proliferation and cytokines production by mitogen-stimulated bovine peripheral blood mononuclear cells

**Published:** 2015-12-15

**Authors:** Reza Norian, Nowruz Delirezh, Abbas Azadmehr

**Affiliations:** 1 *Department of Microbiology, Faculty of Veterinary Medicine, Urmia University, Urmia, Iran; *; 2 *Department of Immunology, Qazvin University of Medical Sciences, Qazvin, Iran.*

**Keywords:** Bovine, Concanavalin A, Cytokine, Phytohemagglutinin, Pokeweed mitogen

## Abstract

This *in vitro* study was conducted to evaluate lymphocyte blastogenic and cytokine production by bovine peripheral blood mononuclear cells (PBMCs) stimulated with phytohemagglutinin (PHA), pokeweed mitogen (PWM) and concanavalin A (Con A) mitogens, by using tetrazolium salt and ELISA tests, respectively. The results presented that Interleukin-2 (IL-2), IL-4, IL-5, IL-10, IL-17 and IFN-γ production in response to PWM mitogens was the highest and Con A the lowest amount and the median values of three mitogens were in the following order: PWM > PHA > Con A > cell control. In the case of IL-6, the production of this cytokine was the same amount for PWM and Con A and a lower amount for PHA stimulation. The results of this study not only showed a normal range for the production of these cytokines from PBMCs that were affected by mitogens, but it demonstrated that the bovine immune system at 2.5 to 3 months was post-natally matured enough to mount an effective immune response to mitogens as well as specific antigens.

## Introduction

The mitogen- induced blastogenic response assay of peripheral blood mononuclear cells (PBMCs) has been widely used to assess non-specific cellular immunity. The most common method for the evaluation of lymphocyte blastogenesis is to quantitate the incorporation of 3H-thymidine during the cultivation of lymphocytes. The 3H-thymidine uptake assay has also been to assess the immunological competence of horses affected with various infectious and immunodeficient diseases.^1^ However, for veterinary clinical studies this method is seldom used, as it requires expensive specialized equipments, and the radioisotopes are needed to be handled in special facilities. A useful colorimetric assay for the measurement of surviving and proliferating cells using a tetrazolium salt, 3-(4, 5-dimethyl thiazol-2-yl)-2, 5-indiphenyl tetrazolium bromide (MTT) has been introduced by Mosmann.^2^

 The MTT assay has been widely applied in various functional tests such as mitogen-induced lymphocyte blastogenesis, cytokine and cytotoxicity assays in human and animals.^3^^,^^4^ The best way to understand true mechanisms of immune responses to pathogens, immune-mediated disorders and mitogens, is the measurement of cytokine production in response to mitogens and specific antigens.^5^^,^^6^ Cytokines are soluble proteins with low molecular weight (5-70 kDa), that are produced and released by individual cells for the purpose of transmitting messages of activation, inhibition, chemo-attraction and apoptosis.^7^ and they are multifunctional molecules that mediate a wide range of physiological responses and their fundamental role is in the immune responses, especially, in normal T-cell-mediated immunity, cancer, autoimmunity, inflammatory responses and allergy.^8^ Mitogens, also called polyclonal lymphocyte activators, are able to induce mitotic proliferation in cells. Their reaction is non-specific, which means that they can influence various lymphocyte subpopulations. The most frequently used mitogens are concanavalin A (Con A), pokeweed mitogen (PWM) and phytohemagglutinin (PHA).^9^ The primary target of their action is the plasma membrane, where they bind to the carbohydrate moiety of membrane glycoproteins. Con A binds to a-D-manosyle, PWM and PHA to N-acetyl- D-glucosamine. The binding of mitogens makes membrane receptors to activate adenylate cyclase (followed by the synthesis of cyclic adenosine monophosphate) or guanylate-cyclase (followed by the synthesis of cyclic guanosine monophosphate), which result in signal transduction from membrane to the nucleus of lymphocytes.^9^* In vitro* stimulation of lymphocytes by respective mitogens makes the cells to produce appropriate cytokines to mount immune response.^10^^,^^11^ Although the number of cytokines is increasing, the normal profile of cytokine production in response to mitogens, especially in calves has not been established for many of the cytokines. 

In this study, in the first stage, we determined the optimal conditions needed for an MTT assay to evaluate the mitogen-induced blastogenic response of bovine PBMCs, and in the second stage, to evaluate the bovine cytokine production by PBMCs *in vitro* and to contribute in understanding of the postnatal development of the immune responses from healthy calves after mitogen stimulation and to determine the normal ranges. In the present study, the following cytokines were measured: Interleukin-2 (IL-2) and interferon-gamma (IFN-γ); IL-4 and IL-5; and IL-17 as representative of Th1, Th2 and Tا17 cytokines respectively, and IL-6 and IL-10 as pro and anti-inflammatory cytokines. 

## Materials and Methods

**Animals.** Fifteen Holstein/Friesian steers, approximately 2.5 to 3 months of age, were included in the present study. All animals were identical in terms of diet, health condition and state of preservation. Blood samples were taken from the jugular vein and were collected in sterile heparin tubes and PBMCs were isolated from whole blood as described below. 

**Media and reagents.** All materials, chemical, reagents and media were obtained from Sigma Chemical Co. (St. Louis, USA), unless otherwise noted. The cell culture medium consisted of RPMI 1640 supplemented with 10% heat-inactivated fetal calf serum (FCS), 10 mM HEPES buffer, 2 mM l-glutamine, 100 U‌ mL^-1^ penicillin, and 100 μgmL^-1^ streptomycin. PBMC were isolated from whole blood by centrifugation (Model 380; Hettich Rotina, Tuttlingen, Germany) through Ficoll-Hypaque solution.

**Optimization of lymphocyte proliferation. **In order to optimize the lymphocyte proliferation assay for use with bovine peripheral blood, various conditions were tested including several T and B cell mitogens such as PWM, PHA and Con A at numerous concentrations (0.5, 1.0, 2.5, 5.0 and 10 μgmL^-1^), multiple incubation time points (24, 48, 72, 96 and 120 hr) and varied cell densities (0.5 × 10^5^, 1 × 10^5^, 2.5 × 10^5^, 5 × 10^5^, 7.5 × 10^5^ and 1 × 10^6^ cells per well). Hence, PBMCs were isolated from whole blood and washed three times in phosphate-buffered saline (PBS; Roche, Paris, France) and re-suspended in culture medium at the varied cell densities listed above. Then, 180 μL of cell suspension was added to wells of a 96-well tissue culture plate in five replicates. Then, 20 μL of mitogens or medium (control) was added to the wells and plates were incubated for five days at 37˚C, 5% CO_2_ and 95% humidity. 

**Cell proliferation and viability assay. **We used MTT assay for evaluation of mitogen-induced blastogenic response of bovine PBMCs. Therefore, 10 μL of MTT (5 μgmL^-1^ of PBS) solution was added to the wells for the last 6 hr of cultivation. At the end of the cultivation, the plates were centrifuged at 400 *g* for 10 min and the culture supernatant was accumulated. A volume of 100 μL of dimethyl sulfoxide (DMSO) was added to each well, after which the plates were vigorously shaken to ensure that all crystals were completely dissolved. The amount of MTT formazan produced during the incubation was measured by an ELISA reader (Model ELx800; Bio-Tek Instruments, Winooki, USA) at a test wavelength of 550 nm and a reference wavelength of 630 nm. The results were based on the optical density at the wavelength of 550 nm (OD_550_) and expressed as a stimulation index (SI), which was calculated as follows:

$\mathrm{SI}=\frac{\mathrm{Mean OD}550\mathrm{of stimulated PBMCs}–\mathrm{Mean OD}550\mathrm{of blank}}{\mathrm{Mean OD}550\mathrm{of unstimulated PBMCs}}$

**Cytokine assays. **Cell culture supernatants were harvested and analyzed for cytokines using bovine IL-2, IL-4, IL-5, IL-6, IL-10, IL-17 and IFN-γ commercially available ELISA kits (all USCN Life Science Inc., Wuhan, China) and the concentration of each cytokine was measured using the ELISA reader at a wavelength of 450 nm .The limits of detection (LOD) for the individual assays were as follow: IL-2, 12.9 pgmL^-1^; IL-4, 6.2 pgmL^-1^; IL-5, 6.2 pgmL^-1^; IL-10, 12.4 pgmL^-1^; IL-17, 14.2 pgmL^-1^; IL-6, 12.6 pgmL^-1^; and IFN-γ, 12.8 pgmL^-1^.

**Statistical analysis. **Statistical analysis was performed using student^’^s *t*-test in SPSS (version 15; SPSS Inc., Chicago, USA) and the results were expressed as mean ± SE values. Significant differences were defined at *p *≤ 0.01.

## Results

**Fig. 1:**
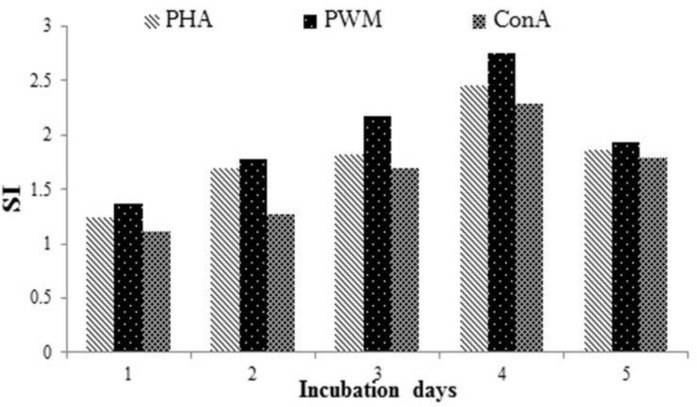
The effect of culture period on blastogenic responses using the MTT assay. PBMCs were cultured at 5 × 10^5^ cells per well with mitogens (Con A at 5.0 μg mL^-1^, PHA at 2.5 μg mL^-1^, PWM at 2.5 μg mL^-1^

**Fig. 2:**
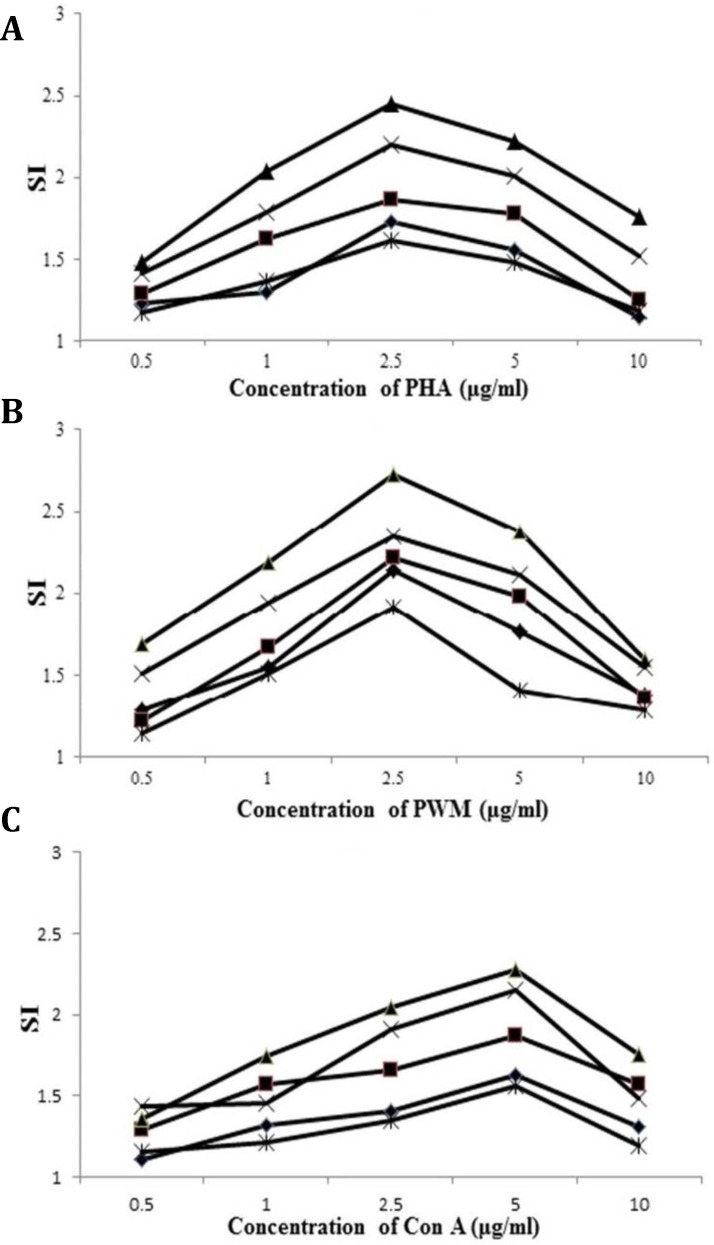
The effect of cell concentrations on blastogenic responses using the MTT assay concentration of PHA (a), PWM (b) and Con A (c). Each point represents the mean of triplicate cultures from fifteen calves.

**Cell proliferation and viability assay. **PBMCs from 15 calves were cultured with various concentrations of mitogens for five days at different cell numbers (Fig. 1). The effects of mitogen concentrations and cell number were determined (Fig. 2). According to results obtained, the optimum concentration of Con A, PHA and PWM mitogens were 5.0, 2.5 and 2.5 μg mL^-1^, respectively. 

The maximum SI values were obtained at 5 × 10^5^ cells per well of cell density for Con A, PHA and PWM stimulation. Also, the optimum incubation period for the appropriate concentration of each mitogen, four days was determined (Fig. 1).

The blastogenic responses of the PBMCs from the healthy calves were examined with three mitogens using MTT test, and the maximal responses were obtained after the PBMCs had been cultured with three mitogens for four days. 

**Cytokine assay. **In the present study, PBMCs from healthy calves were stimulated with conventional mitogens and the cytokines produced were measured in super-natant of the culture. In general, we found a wide range of values for each mitogen, therefore, the median rather than the mean values were considered more indicative.

**Fig. 3:**
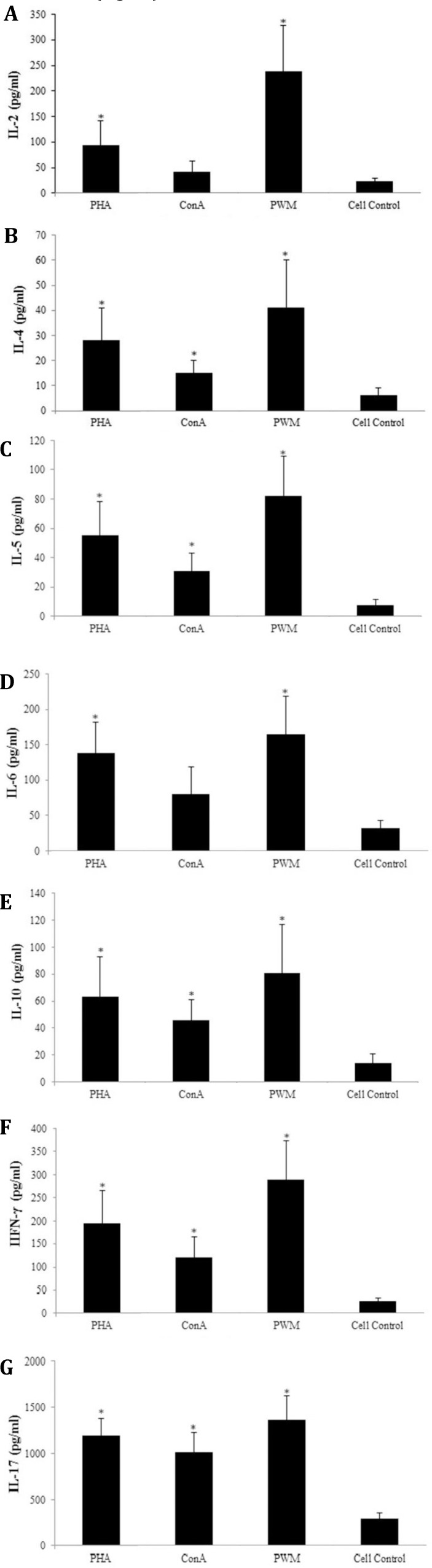
Cytokine production by PBMCs, from healthy calves after four days stimulation with PMW, PHA and Con A mitogens, as determined by ELISA. (A) for IL-2, (B) for IL-4, (C) for IL-5, (D) for IL-6, (E) for IL-10, (F) for IFN-γ, (G) for IL-17.

**Interleukin-2.** IL-2 production in response to mitogens showed a wide range of values, as PWM mitogen had more effective role in stimulating the PBMC cells in IL-2 synthesis when compared to other mitogens. The median values of IL-2 production due to the mitogen stimulatory effects, demonstrated the following order: PWM > PHA > Con A > cell control (Fig. 3A).

**Interleukin-4. **The range of values ​​of‌ these cytokines were narrower than other cytokines. The order of preferential stimulation was PWM > PHA > Con A > cell control (Fig. 3B).

**Interleukin-5. **The results demonstrated a close range of values. The order of preferential stimulation was PWM > PHA > Con A > cell control (Fig. 3C).

**Interleukin-6. **The results of IL-6 production showed a wide range of values. In this case, as in other studies, PWM and PHA were much more effective in generating IL-6 synthesis than the Con A mitogen. The order of preferential stimulation was PWM > PHA > Con A > cell control (Fig. 3D).

**Interleukin-10. **According to the results obtained, the order of preferential stimulation was PWM > PHA > Con A > cell control (Fig. 3E).

**Interferon-gamma. **The results of production of IFN-γ are demonstrated a wide range of values. The order of preferential mitogen stimulation was PWM > PHA > Con A > cell control (Fig. 3F).

**Interleukin-17.** The results of IL-17 production revealed a broad range with all three mitogens and the cell control. According to the results obtained, the order of preferential mitogen stimulation was PWM > PHA > Con A > cell control (Fig. 3G).

▲: 5 ×10^5^ cells per well, **×**: 1 × 10^6^ cells per well, ■: 2 × 10^5^ cells per well, ♦: 1 × 10^5^ cells per well, *: 2 × 10^6^ cells per well.

* indicates the significant differences between mitogen stimulated and control cells *p* ≤ 0.05.

According to the results presented above IL-2, IL-4, IL-5, IL-10, IL-17 and IFN-γ production in response to PWM mitogens was the highest and Con A as the lowest amount and the median values of three mitogens were in the following order: PWM > PHA > ConA > cell control. In the case of IL-6, the production of this cytokine was the same amount for PWM and Con A and a lower amount for PHA stimulation.

## Discussion

The PHA, Con A and PWM are potent mitogens which were used for leukocyte proliferation and differentiation most frequently.^5^^,^^11^ Because of their non-specific effects, it was not possible to evaluate the response of individual bovine cells that produced cytokines until recently, when the ELISA and ELISpot kits for bovine mononuclear leukocytes became available.

This study implied that the bovine immune system at that age was matured enough to produce enough response to mitogenic stimuli and also determined normal ranges for bovine IL-2, IL-4, IL-5, IL-6, IL-10, IL-17 and IFN-γ levels. In agreement with other authors, it was shown, that PWM had a stronger mitogen activity compared to PHA and Con A.^12^

It is known that PWM has two specific domains available for binding to membrane receptors,^13^ and therefore has a higher potential for its action. This may explain the fact that the mitogenic effect of PWM on mononuclear leukocytes was higher than that of PHA and Con A. In other words, PWM stimulated T cells, particularly helper T cells and B cells.^5^^,^^14^ In comparison with PWM, PHA had similar effects, however, was less powerful in stimulating of B cells. Con A is reported to stimulate T cells, particularly cytotoxic T cells,^15^ suppressor inducer T cells,^16^ and “virgin” T cells.^15^

In this study, PBMCs were stimulated with these mitogens and the cytokines produced were measured in supernatant of the culture. The final concentrations for Con A, PWM and PHA were 5.0, 2.5 and 2.5 μgmL^-1^, respectively, and the optimum incubation period for the appropriate concentration of each mitogen, four days was determined.

Tajima reported that an incubation of cells for 72 hr with PHA, and 96 hr with Con A or PWM was a suitable time period for equine PBL blastogenic response using ethidium bromide (EB). Sanada reported that the optimum incubation period of equine PBL was 120 hours with all three mitogens using results of the EB.^17^

The IL-2 is necessary for the growth, proliferation and differentiation of T cells to become 'effector' T cells. It is produced by T cells during an immune response to antigen. The high levels of IL-2 production by PBMC in response to mitogens may be due to the greater role of T cells in response to mitogen and its key role in regulating of the immune response.

The IL-4 is produced by activated T cells, eosinophils, mast cells and basophils, and induces differentiation of naive helper T cells to T Helper 2 (Th2) cells, and is a key regulator in humoral and adaptive immunity. The IL-4 stimulates production of antibody-producing B cells, leading to the production of immunoglobulin (Ig), and class-switching to IgE in allergic disease. Increased IL-4 levels indicate an elevated activity of eosinophils and mast cells. The range of our values for IL-4 was low and may be due to the non-allergic state of calves.

The IL-5 produced by Th2 and mast cells played a specific role in the control of eosinophil production and differentiation, therefore, in a healthy donor; one would not expect the lymphocytes to be primed to produce increased IL-5 levels. This would explain the close range noted for IL-5 production. 

The IL-6 is produced by a variety of cell types. The principal cell sources for IL-6 are macrophages, fibroblasts, endothelial, T and B cells. The IL-6 is pleiotropic (multi-functional) and acts as both pro-inflammatory and anti-inflammatory cytokine. It is one of the key mediators of fever and of the acute phase response. Furthermore, the high levels of IL-6 obtained as a result of stimulation may be due to preferential stimulation of monocytes/macrophages, B and T ‌cells.

The IL-10 is an anti-inflammatory cytokine produced by activated monocytes, T and B cells. The IL-10 is involved in the function of a number of cells, and influences many physiological processes, including angiogenesis, tumor genesis, and infection. The IL-10 down-regulates the expression of Th1 cytokines and is an important B cell and mast cell growth factor. The relativ values of IL-10 observed may have been due to the stimulation of monocytes and Th2 cells.

The IL-17 is a recently described cytokine produced exclusively by CD4 helper/inducer T cells upon activation, most often acting as a pro-inflammatory cytokine that stimulates the release of secondary cytokines and chemokines. The IL-17 induces the recruitment of neutrophils for antimicrobial effects. The relatively high values of IL-17 observed, may be due to T cell responses to the mitogens (PWM, PHA, and Con A), demonstrating that T cell-specific signaling is involved in the production of IL-17. There is also direct correlation between production of IL-6 and IL-17. Recent works from several groups indicated that IL-6 and transforming growth factor-β (TGF-β) potently initiated Th17 differentiation.

The IFN-γ is produced mainly by activated lymphocytes (T cells and NK cells). IFN-γ regulates cellular activities responsible for inflammation. It further modulates the antigen specific immune response by affecting both APCs and antigen-recognizing lymphocytes; IFN-γ also promotes innate and adaptive immune responses in the host against variety of infectious agents, tumors, trauma, and autoimmune diseases. The large scale values of IFN-γ observed may be due to the stimulation of T cells by mitogens.

The increased levels of IL-2, IL-4 and IL-5 noted secondary to PWM and somewhat PHA may be due to dual stimulation of both T and B lymphocytes. The B cells may produce an amplification factor which then stimulates the T lymphocytes to increase IL-2, IL-4 and IL-5 production. Also to increase the levels of IL-6 and IL-17 noted for all T lymphocyte stimulators for example PHA, Con A and PWM mitogens. Because these cytokines produced from activated T lymphocytes and increased secretion of IL-6, lead to an increase in production of IL-17.

This study assessed whether a pattern existed between cytokine levels for each individual for a given mitogen. The PHA and PWM results were consistent with Th1 and Th2 profiles, respectively.

 For all three mitogens (PHA, PWM and Con A), a relationship existed between IL-2 and IFN-γ levels.‌ Stimulation with PWM also showed the same relationship between IL-4 and IL-5, and between IL-6 and IL-17.

Con A showed that unlike to other mitogens (PWM, PHA), it was less effective in stimulating of PBMCs to cytokine production, although it is possible in many cases, that it would not be corroborated with the classic Th1-Th2 paradigm.^18^^,^^19^

The majority of the literature has described cytokines such as IL-2 as being produced by Th1 cells. However, this was originally described for the murine model. Borish and Rossenwasser demonstrated that the breakdown of the Th1 and Th2-cell cytokines in humans is not as clearly divided.^8^ For example, IL-2 is produced by both Th1 and Th2 lymphocytes. The cytokine associations noted in this study are very interesting, and most of them appear to fit the Th1, Th2 and Th17‌ paradigm. However, this was not the primary objective of this study, and thus further investigation is needed to delineate the direct and inverse relationships between cytokines in cattle. Previous studies revealed that PBMCs from chronic mucocutaneous candidiasis patients produced more IL-4, but not IL-10, IL-2R, or IFN-γ, in response to PHA than PBMCs from controls and PBMCs from foot-and-mouth disease virus (FMDV) vaccinated pigs that produced more IL-2, IL-4, IL-6, IL-10 and IFN-γ in response to FMDV than PBMCs from controls.^20^^,^^21^ In conclusion, in the present study, we found that PBMCs from healthy calves produced variety amounts of pro or anti-inflammatory cytokines in response to PWM, Con A and PHA mitogens, which may be used as an indicator to evaluate cytokine production in clinical as well as research settings.

## References

[1] Furr MO, Crisman MV, Robertson J (1992). Immuno-deficiency associated with lymphosarcoma in a horse. J Am Vet Med Assoc.

[2] Mosmann T (1983). Rapid colorimetric assay for cellular growth and survival: Application to proliferation and cyto-toxicity assays. J Immunol Methods.

[3] Iwata H, Inoue T (1993). The colorimetric assay for swine lymphocyte blastogenesis. J Vet Med Sci.

[4] Inokuma H, Yoshida T, Onishi T (1995). Development of peripheral blood mononuclear cell response to mitogens in Japanese black newborn calves. J Vet Med Sci.

[5] Zhao S, Zhao X, Su H (2010). Development of MTT assay for the detection of peripheral blood T cell proliferation of swine. Chin Anim Husbandry Vet Med.

[6] Mäkitalo B, Andersson M, Areström I (2002). Elispot and Elisa analysis of spontaneous, mitogen-induced and antigen-specific cytokine production in cynomolgus and rhesus macaques. J Immunol Methods.

[7] Murtaugh MP (1994). Porcine cytokines. Vet Immunol Immunopathol.

[8] Borish L, Rosenwasser LJ (1996). Update on cytokines. J Allergy Clin Immunol.

[9] Wimer BM (1996). Putative effects of mitogenic lectin therapy corroborated by allo-activation data. Cancer Biother Radiopharm.

[10] Wattrang E, Palm AK, Wagner B (2012). Cytokine production and proliferation upon in vitro oligodeoxyribonucleotide stimulation of equine peripheral blood mononuclear cells. Vet Immunol Immunopathol.

[11] Zhang BB, Zhao K, He WQ (2011). Study on optimal condition of MTT in PBMC transformation. Progress Vet Med.

[12] Chen XD, Lei T, Xia T (2004). Increased Expression of resistin and tumour necrosis factor-alpha in pig adipose tissue as well as effect of feeding treatment on resistin and Camp pathway. Diabetes Obes Metab.

[13] Symons DB, Lay CA, MacDonald AN (1977). Stimulation of pig lymphocytes with anti-immunoglobulin serum and mitogens. Int Arch Allergy Appl Immunol.

[14] Miller RA, Flurkey K, Molloy M (1991). Differential sensitivity of virgin and memory T lymphocytes to calcium ionophores suggests a buoyant density separation method and a model for memory cell hypo-responsiveness to Con A. J Immunol.

[15] Simon MM, Hochgeschwender U, Brugger U (1986). Monoclonal antibodies to interferon-gamma inhibit interleukin 2-dependent induction of growth and maturation in lectin/antigen-reactive cytolyticT lymphocyte precursors. J Immunol.

[16] Morimoto C, Letvin NL, Boyd AW (1985). The isolation and characterization of the human helper inducer T cell subset. J Immunol.

[17] Sanada Y, Noda H, Nagahata H (1990). Changes in lymphocyte blastogenic response of mares during the perinatal period. Jpn J Vet Sci.

[18] Tajima M, Fujinaga T, Koike T (1987). Assay for Equine peripheral blood lymphocytes blastogenic response using ethidium bromide. Jpn J Vet Sci.

[19] Kobrynski LJ, TanimuneL, Kilpatrick L (1996). Production of T-helper cell subsets and cytokines by lymphocytes from patients with chronic mucocutaneous candidiasis. Clin Diagn Lab Immunol.

[20] Barnard AL, Arriens A, Cox S (2005). Immune response characteristics following emergency vaccination of pigs against foot-and-mouth disease. Vaccine.

[21] Al-Janadi M, Al-Balla S, Al-Dalaan A (1993). Cytokine profile in systemic lupus erythematosus, rheumatoid arthritis, and other rheumatic diseases. J Clin Immunol.

